# Study of the Lipolysis Effect of Nanoliposome-Encapsulated *Ganoderma lucidum* Protein Hydrolysates on Adipocyte Cells Using Proteomics Approach

**DOI:** 10.3390/foods10092157

**Published:** 2021-09-12

**Authors:** Sucheewin Krobthong, Yodying Yingchutrakul, Wonnop Visessanguan, Thanisorn Mahatnirunkul, Pawitrabhorn Samutrtai, Chartchai Chaichana, Phakorn Papan, Kiattawee Choowongkomon

**Affiliations:** 1 Interdisciplinary Graduate Program in Genetic Engineering, Kasetsart University, Bangkok 10900, Thailand; sucheewin82@gmail.com; 2 National Omics Center, NSTDA, Pathum Thani 12120, Thailand; yodying.yin@nstda.or.th; 3 National Center for Genetic Engineering and Biotechnology, NSTDA, Pathum Thani 12120, Thailand; wonnop@biotec.or.th; 4 National Nanotechnology Center, NSTDA, Pathum Thani 12120, Thailand; thanisorn.mah@nanotec.or.th; 5 Department of Pharmaceutical Sciences, Faculty of Pharmacy, Chiang Mai University, Chiang Mai 50200, Thailand; pawitrabhorn.s@cmu.ac.th; 6 Siriraj Center of Research Excellence for Diabetes and Obesity (SiCORE-DO), Faculty of Medicine Siriraj Hospital, Mahidol University, Bangkok 10700, Thailand; chartchai.chi@mahidol.ac.th; 7 Department of Chemistry, Faculty of Science, Chiang Mai University, Chiang Mai 50200, Thailand; phakorn_papan@cmu.ac.th; 8 Department of Biochemistry, Faculty of Science, Kasetsart University, Bangkok 10900, Thailand; 9 Center for Advanced Studies in Nanotechnology for Chemical, Food and Agricultural Industries, KU Institute for Advanced Studies, Kasetsart University, Bangkok 10900, Thailand

**Keywords:** RSM, Lingzhi, hypolipidemic activity, peptides, 3T3-L1

## Abstract

Excessive lipid accumulation is a serious condition. Therefore, we aimed at developing safe strategies using natural hypolipidemic products. Lingzhi is an edible fungus and potential lipid suppression stimulant. To use Lingzhi as a functional hyperlipidemic ingredient, response surface methodology (RSM) was conducted to optimize the time (X_1_) and enzyme usage (X_2_) for the hydrolysate preparation with the highest degree of hydrolysis (DH) and % yield. We encapsulated the hydrolysates using nanoscale liposomes and used proteomics to study how these nano-liposomal hydrolysates could affect lipid accumulation in adipocyte cells. RSM analysis revealed X_1_ at 8.63 h and X_2_ at 0.93% provided the highest values of DH and % yields were 33.99% and 5.70%. The hydrolysates were loaded into liposome particles that were monodispersed. The loaded nano-liposomal particles did not significantly affect cell survival rates. The triglyceride (TG) breakdown in adipocytes showed a higher TG increase compared to the control. Lipid staining level upon the liposome treatment was lower than that of the control. Proteomics revealed 3425 proteins affected by the liposome treatment, the main proteins being TSSK5, SMU1, GRM7, and KLC4, associated with various biological functions besides lipolysis. The nano-liposomal Linzghi hydrolysate might serve as novel functional ingredients in the treatment and prevention of obesity

## 1. Introduction

Modern functional food products are available on the market, ranging from isolated nutrients, dietary supplements, and specific products to processed or engineered foods. Peptides from foodstuff are candidates for functional food ingredients due to their beneficial health aspects such as immune-boosting, anti-oxidative stress, hypolipidemic and tumor suppressing activity [1,2]. One of the above-mentioned beneficial aspects is the hypolipidemic activity on adipocytes, affecting lipid storage, directly associated with obesity, a contemporary health problem. Obesity is caused by excessive triacylglycerol (TAG) accumulation in the adipocytes. Increasing TAG breakdown or hypolipidemic activity might contribute to reducing body fat and triglyceride (TG) levels. Several natural-sourced peptides could be combined with foodstuffs, and their effective delivery could display beneficial aspects [3].

Most edible mushrooms such as *Volvariella volvacea*, *Lentinula edodes*, and *Ganoderma lucidum* are beneficial for health. They have been generally consumed as basic food, as they provide plenty of dietary nutrients including fibers, minerals, and vitamins. They are also excellent sources of proteins [4]. Beyond their role as foodstuffs, edible mushrooms feature in certain types of holistic or alternative medicine. *G. lucidum*, locally known as Lingzhi, is defined as a medicinal mushroom for the prevention of various diseases, as well as for recuperation and health improvement. Ganoderma species are generally found all over the world. Lingzhi exhibits the prevailing features of being an excellent nutrient source of proteins, lipids, and carbohydrates [5]. Lingzhi has been consumed widely in East-Asia as a traditional remedy for centuries [6]. Many of its pharmacological effects have been widely reported such as immune modulation enhancement, soothing the nerves, inflammatory response reduction, cancer growth suppression, cell-aging deceleration, oxidative stress reduction, and anti-aging and lipid accumulation suppressive effects [7,8,9].

Foodstuff-derived protein hydrolysates contain a high level of functional peptides. The dominant features of the protein hydrolysates are their lower molecular weight and relative lack of high-order structure, as well as the increased number of functionally ionizable and exposed hydrophobic groups compared to those of intact proteins. These features denote that their surface interactions, water-solubility, host-receptors, and biological activities might be different from those of proteins. This includes the transduction triggering capability of various signaling pathways, leading to the activation or deactivation of regulators and biological activities above their generic nutritional value [10]. However, the major obstacle in introducing peptides into functional food ingredients is their functional stability during commercial processing and under human physiological conditions [11]. Therefore, functional peptides might partially or completely lose their activity before reaching the target cells or organs [12]. Hence, choosing a delivery system that is highly compatible with human physiological conditions would alleviate this problem.

Liposome encapsulation is a well-known compatibility delivery approach for foodstuff hydrolysates. The advantage of encapsulation within small particles is the stability and bioactivity enhancement of the protein hydrolysates [13]. This approach is suitable for protein and peptide delivery as their molecules possess various polar and non-polar regions similar to their liposome properties [14]. Several studies revealed the potential of liposomal encapsulation of peptides. For example, a pharmacological study of ghrelin, the appetite-stimulating peptide hormone, indicated increasing ghrelin stability and circulation period in the blood [15]. Both the pharmaceutical and cosmetic industries generally use liposome-based carriers to store and deliver functional proteins and peptides for specific purposes [16,17]. Although the use of liposomal encapsulation can be observed in a small number of products in the food industry market, liposomal encapsulation would be a promising approach as its safety and efficiency are proven by the pharmaceutical and food industries.

In this study, we established liposome carriers for protein hydrolysates to enhance the biological activities and stability of the latter. In addition, we also investigated the lipolysis-stimulating activity of the encapsulated Lingzhi protein hydrolysates on 3T3-L1 adipocyte cells. A possible signaling pathway for the encapsulated hydrolysates on the stimulation of lipid breakdown was also investigated using quantitative proteomic analysis. Finally, the possible beneficial mechanisms of the nano-liposomal hydrolysates are clarified and their value as a functional food additive supported.

## 2. Materials and Methods

### 2.1. Ganoderma Lucidum Hydrolysate Preparation

Dried Lingzhi (200 g) was powdered using an ultra-centrifugal mill (Retsch Co., Haan, Germany) equipped with a sieve (diameter = 1 mm^3^) at 8000 rounds per minute (rpm). The powdered mushroom was heated using a modified Pressurized Hot Water Extraction method [18]. Briefly, the Lingzhi was mixed with deionized water at a ratio of 1:2 (*w*/*v*) and incubated at 121 °C, 15 psi for 20 min. The extracted Lingzhi was left to cool down and hydrolyzed with pepsin as the first independent factor (X_1_) at 0.25%, 0.5%, and 1% in 0.1% of HCl for digestion times of 3, 6, and 9 h as the second independent factor (X_2_) at a constant temperature of 37 °C. Next, the crude was filtrated with a 0.22-µm nylon membrane and fractionated through Vivaspin-20 (GE Healthcare Co., Amersham, UK), with a molecular weight cut-off of 3 kDa. Peptides of <3 kDa were subjected to Solid-Phase Extraction (SPE) (Waters Co., Milford, MA, USA). An amount of 3 mg of small peptides was loaded on an equilibrated SPE column (Sep-Pak C18) and eluted using acetonitrile: water (1:1, *v*/*v*). The supernatant was dried using a freeze-drying machine.

### 2.2. Lingzhi Protein Hydrolysate Optimization by Response Surface Methodology (RSM)

The two independent variable factors used in this study were the digestion time (X_1_) and the enzyme concentration (X_2_). The experimental outputs were the degree of hydrolysis (DH) (Y_1_) and the product yield (Y_2_). The DH determination was performed according to the method of Nielsen et al. [19] and the product yield was calculated as a percentage of the proteins found in the hydrolysates divided by the raw protein content. While calculating the optimal condition of an independent factor, the values of the other independent factors were fixed. An experimental design was set with 11 conditions, including 9 experimental conditions and 2 central points. The correlation of the independent factors and experimental outputs was used to generate RSM by the following equation:

(1) $$y=\beta_{0}+\varepsilon +\overset{k}{\underset{i=1}{\sum }}\beta_{i}x_{i}+\overset{k}{\underset{i=1}{\sum }}\overset{k}{\underset{j=1}{\sum }}\beta_{ij}x_{i}x_{j}+\overset{k}{\underset{i=1}{\sum }}\beta_{ii}x_{ij}^{2}$$

where *y* is the experimental output; *β*_0_ is constant intercept value; *β_i_*, *β_ii_*, and *β_ij_* are the linear, quadratic, and interaction coefficients, respectively; and *x_i_* and *x_j_* are the independent variable factors. Three-dimensional response surface plots were drawn to illustrate the correlation between the levels of the process variable factors and the outcome results.

### 2.3. Nano-Liposome Carrier Preparation and Characterization

Soybean lecithin (Sigma Aldrich Co., St. Louis, MO, USA) and cholesterol (Sigma Aldrich Co., St. Louis, MO, USA) (8:1, *w*/*w*) were dissolved in 10 mL of diethyl ether in a 50-mL round bottom flask for 5 min. Once the lipids were thoroughly mixed in diethyl ether, the solvent was removed to yield a lecithin-cholesterol film layer by rotary evaporation (Buchi Co., Flawil, Switzerland) at 100 rpm under reduced pressure. The hydration of the lecithin-cholesterol film layer was accomplished by adding 10 mL of Lingzhi extract and agitating on an orbital shaker at 220 rpm for 6 h at 28 °C to obtain a vesicular white suspension. The vesicular suspension was forced through a membrane filter with a defined pore size of 200 nm by an extruder (GE Healthcare Co., Amersham, UK). After day 7, the loading efficiency of the loaded nanoliposome was determined by a protein-based spectrophotometric analysis. We mixed 100 µL samples of loaded liposomes with 1% Triton X-100 (Sigma Aldrich Co.) and sonicated for 10 min (10 s-interval) to disassemble the liposomes and release the extract. Afterward, the protein content of the clearance solution was assessed by Lowry protein assay using Bovine Serum albumin (Sigma Aldrich Co.) as a reference. The loading efficiency was calculated using the following equation:

Extracted loading Efficiency (*w*/*w*) (%) = (protein extracted of which encapsulated in

liposomes (mg) ÷ protein content of extracted Lingzhi (mg)) × 100(2)

The hydrodynamic diameter of the liposomal formulations in deionized water was measured by dynamic light scattering (DLS) using ZetaSizer Nano-ZS (Malvern Instruments, Worcestershire WR, UK), in which the zeta potential was also examined (*n* = 3).

### 2.4. Effect of Loaded Nanoliposomes on 3T3-L1 Adipocyte Cells

Cell cytotoxicity of the loaded liposome and unloaded liposome control was evaluated through an MTT assay. Human fibroblasts (American Type Culture Collection., Manassas, VA, USA)) and 3T3-L1 mouse differentiated adipocyte cells (induced by an adipogenic cocktail containing 2.5 mM dexamethasone, 0.5 mM 3-isobutyl-1-methylxanthine, and 10 g/mL insulin for 8 days) were tested for cytotoxicity at various concentrations (104.68, 52.34, 26.17, 13.09, 6.54, 3.27, 1.64, 0.82, 0.41, and 0.20 µg/mL) of loaded liposomes and unloaded liposome as control for 24 h. Next, we measured the optical absorbance at 570 nm using a microplate reader and transformed the results into cell survival rate percentage [20].

The lipolytic effect of the loaded nanoliposome was used to quantify glycerol, a byproduct of lipolysis (EnzyChrom™ Glycerol Assay Kit, BioAssay Systems, Hayward, CA., USA) in cell culture supernatant after 24 h of treatment with the loaded nanoliposome. To determine the intracellular TG content, the differentiated 3T3-L1 cells were treated with the loaded nanoliposomes, as described previously, for 24 h. The cells were washed twice with PBS and fixed with 4% paraformaldehyde for 1 hour at room temperature. Next, the cells were washed once with PBS and isopropanol 60% (*v*/*v*), then they were allowed to dry. Next, the cells were stained with 0.5% (*v*/*v*) Oil Red O (ORO) (Sigma Aldrich Co.) in an isopropanol solution of 60% for 1 hour. After staining, the unstained dye was removed by rinsing with distilled water. The stained lipid droplets were observed under a stereomicroscope. The stained oil droplets indicating lipid accumulation were solubilized by absolute isopropanol for 15 min and their absorbance was measured at 510 nm using a microplate reader (Multiskan Go, Thermo Scientific, Waltham, MA, USA).

### 2.5. Proteomic Analysis and Data Processing

To investigate the adipocyte protein expression profiles after the exposure to the loaded liposomes, the cells were lysed by a lysis buffer solution (10 mM HEPES-NaOH pH 8.0 and 0.5% Triton X-100) supplemented with a protease inhibitor cocktail (Thermo Scientific Co.). The supernatant was collected by centrifugation, followed by ice-cold acetone precipitation (1:5 *v*/*v*). After precipitation, the protein pellet was reconstituted in 0.2% RapidGest SF (Waters Co.) in 10 mM of Ammonium bicarbonate (Sigma Aldrich Co.). The total protein (50 µg) was subjected to gel-free based digestion. Next, sulfhydryl bond reduction was performed using 5 mM DTT (Sigma Aldrich Co.) in 10 mM ammonium bicarbonate at 72 °C for 1 h and sulfhydryl alkylation using IAA (Sigma Aldrich Co.) at room temperature for 30 min in the dark. The solution was cleaned up using a Desalting Zebra-spin column (Thermo Scientific Co.). The flow-through solution was enzymatically digested by Trypsin (Promega Co., Madison, WI, USA) at a ratio of 1:50 (enzyme: protein) and incubated at 37 °C for 3 h. The digested solution was dried and reconstituted in 0.1% formic acid before being subjected to tandem-mass spectroscopy using a nanoLC-system coupled with high resolution 6600 TripleTOF^TM^ (AB-Sciex, Concord, ON, Canada). The LC conditions were as follows: mobile phase A and B were used, with mobile phase A being composed of 0.1% formic acid in water and mobile phase B comprising 95% acetonitrile with 0.1% formic acid. The LC-method parameters comprised a 135-min long process for a single injection. The analytical column was maintained at 55 °C. Using the data-dependent acquisition mode of mass spectroscopy, the MS scans over a mass range of 400–1600 *m/z*, selecting the top 20 most abundant peptide ions with charge state in the range of 2–5 (positive mode) for fragmentation. The dynamic exclusion duration was set at 15 s. The raw MS-spectra resulting (.wiff) file was extracted and annotated with protein sequences using the Paragon™ Algorithm by ProteinPilot™ Software [21]. The *Mus musculus* protein database, retrieved from UniProtKB (16,477 sequences) and used in Paragon™, was assembled in FASTA format and downloaded in May 2021. We set a detected protein threshold of (Unused ProtScore (Conf)) ≥  0.05 with 1% false discovery rate (FDR) with ≥10 peptides/protein. The protein and peptide comparisons exhibiting >20% coefficient of variation (C.V.) between the replicates were rejected. Both library and SWATH-MS data were imported into SWATH^TM^ processing microapp in PeakView^®^ software. The normalization of the relative protein abundances was performed using the R package, NormalyzerDE [22], in which Quantile-normalization was applied to expression data analysis, after adding 1 to all expression values to avoid errors upon log transformation.

### 2.6. Statistical Analysis

All experiments were carried out in at least three independent replicates (*n* = 3), and all data were expressed as the means ± standard deviation. The statistical significance was determined by Duncan’s multiple range test (*p*-values < 0.05). For the RSM analysis, the generated 3D surface was determined from the fitted polynomial equation, and significant coefficients (*p* < 0.01) were used in the model. The variance table was generated from both independent variables and experimental outputs using the Design Expert statistical software (version 11.0; State-Ease Inc., Minneapolis, MN, USA). For the pairwise comparisons during the proteomic analysis, we performed One-Way analysis of variance (One-Way ANOVA) at the protein-level analysis with two multiple testing correction methods including the Bonferroni and the Benjamini–Hochberg FDR corrections using the ProteinPilot™ Software.

## 3. Results

### 3.1. Lingzhi-Derived Protein Hydrolysate Optimization

The biological activity of the hydrolysates depends on the processing conditions. The activities of various foodstuff hydrolysates were reportedly directly dependent on the degree of hydrolysis, protease activity, and amino acid arrangement [23]. The optimum conditions for the Lingzhi hydrolysate regarding DH and product yield for functional food product manufacturing have not yet been established. Therefore, the present study was aimed at Lingzhi hydrolyzing proteins using RSM to study the effect of the processing conditions including time, enzyme usage on DH, and product yield of the resulting hydrolysates. We applied quadratic analysis statistics to fit an RSM model for independent variable factors. The experimental design using two independent variable factors with two center points (experiment no. 10 and 11) in RSM generation resulted in the observed DH and yield as displayed in Table 1. The RSM generation-related statistical value is shown in Appendix A.

As outputs from the overall experimental design, the DH and product yield ranged from 28.11% ± 1.03% to 34.18% ± 1.12% and 4.16% ± 0.13% to 5.70% ± 0.20%, respectively. The difference in the DH and yield could be due to the difference in the digestion time and enzyme concentration. The equation for multiple regression analysis during the RMS was performed to resolve the coefficients of the independent factors of the linear (x_1_, x_2_), quadratic (x_1_^2^, x_2_^2^), and two-factor relation (x_1×2_) to fit the RSM. According to the multiple regression analysis, the explanatory model equation of the DH (y_1_) and percentage of product yield (y_2_) is given as follows in Table 2.

**Figure 1:**
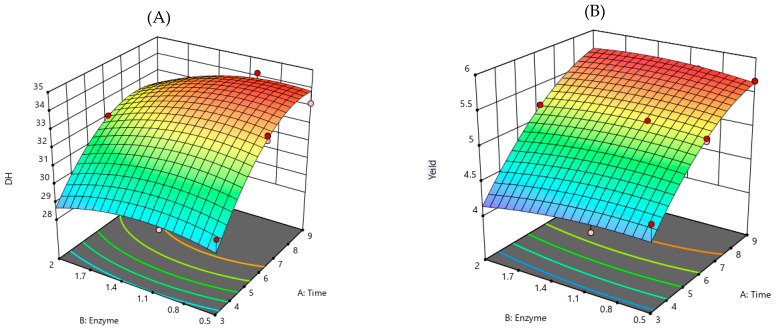
3D-RMS plots showing the interactive effects of different factors on DH and yield. (**A**) DH of Lingzhi protein hydrolysate on digestion time versus enzyme usage, and (**B**) yield of Lingzhi protein hydrolysate on digestion time.

The total coefficient value (R^2^) was used to imply the model suitability. The R^2^ of the DH and the product % yield were 0.958 and 0.968, respectively. This result indicated that the variation in the experimental data was lower than 5% (within 95% level of confidence). The 3-dimensional response model surfaces (3D-RMS) for each variable are illustrated in Figure 1.

The experimental outputs of the processing related to both independent factors, DH (Figure 1A) and % yield (Figure 1B) indicating that the hydrolysate processing depended on the digestion time and enzyme usage. The 3D-RMS for the DH of hydrolysate as a function of digestion time, at fixed enzyme usage, revealed that DH was dependent on the digestion time. Also, DH increased with enzyme usage at the fixed digestion time, suggesting that DH was also dependent on the enzyme usage. Yield also had correlative results, dependent on the digestion time and enzyme usage. In order to obtain the highest DH and product yield, the RSM model was optimized by setting the highest value of response variable factors. As a result, X_1_ was 8.63 h and X_2_ was 0.93%, and the highest values of y_1_ and y_2_ were 33.99% and 5.70%, respectively. These characteristics of DH and yield curves were associated with feedback inhibition during the hydrolysis, where products may act as an inhibitor to protease [24]. The curves strongly suggested that the processing at different conditions and factors were involved. The independent factors, both time and enzyme concentration, had the optimum range for hydrolysate production to gain the maximum DH and yield. To endorse the reliability and validity of the model for processing, the assays were performed under those optimal conditions. The actual experimental values for DH and product yield were 32.71 ± 0.17% and 5.44 ± 0.14%, respectively; the experimental values fitted with the values that were predicted by the model within a 95% confidence interval. These results confirmed that the model was suitable for Lingzhi protein hydrolysate processing for use as functional ingredients regarding cost- and time-efficiency.

### 3.2. Encapsulation Efficiency and Loaded Liposome Size, Polydispersity Index, and Zeta Potential

The encapsulation efficiency of the liposomal formulation was estimated. The liposomes would passively entrap the protein hydrolysate in their hydrophilic region. However, many factors influence the entrapping efficiency such as lipid molar ratios, molecular size, charge, and molecule stability. To evaluate the entrapping efficiency, we used a non-ionic detergent, Triton X-100, as a neutral detergent to disrupt the liposome shell structure, thereby allowing the leakage of the encapsulated Lingzhi protein hydrolysate [25]. Based on the encapsulation condition, 61.24 ± 3.18% of the encapsulation efficiency was achieved. The encapsulation efficiency showed that the liposomal preparation for protein hydrolysate moderates the encapsulated level. The protein hydrolysate has a mixture of peptides with a variety of molecular weights, sizes, charges, and structures. Middle-sized peptides might interact with the lipid layer and form an oligomerization structure like a beta-barrel. This could disrupt the entrapped protein hydrolysate inside the core structure of the liposome [26]. Another reason was the fluctuation in electrostatic interaction between the charges of various peptides and the liposome surface, which might negatively affect the encapsulation efficiency.

The diameter of the nanoliposome in the closest realistic physiological condition was determined. Dynamic light scattering (DLS) analysis showed loaded liposome diameters in the PBS solution were at 149.84 ± 0.58 nm (Figure S1). Low polydispersity index (PdI) of =0.048 ± 0.014 supported that particles were monodispersed. In addition, the low PdI value also reflected that the particle exhibits a narrow size distribution, providing a very high surface area that would be ideal for the correct order. This evidence suggested the homogeneity of the loaded liposome. The overall charge of loaded liposomes was neutral. Zeta (ζ)-potential of the loaded liposome was −3.75 ± 0.25 mV (Figure S2). This could suggest that the overall structure of the liposome exhibited neutral charge particle, due to the value of ζ-potential ranging from −10 to +10 mV, is considered neutral [27]. The hydrodynamic size of the loaded liposome was roughly 140 nm, indicating that the liposome was characterized in the nanoscale. As the efficiency of cellular uptake relates to the particle size, a small particle size of around 100–160 nm would have great potential for cellular uptake into the blood steam via clathrin-dependent mechanisms [28]. Beneficial properties of the negative value of ζ-potential were particle stability under physiological conditions and the prevention of cellular fusion and aggression of phagocytosis, responding less than the positive value of ζ-potential [29]. Therefore, the hydrodynamics of loaded liposome size and negative ζ-potential are the two key criteria that have been considered for various applications.

### 3.3. Effect of Loaded Nanoliposome on 3T3-L1 Adipocyte Cells

**Figure 2:**
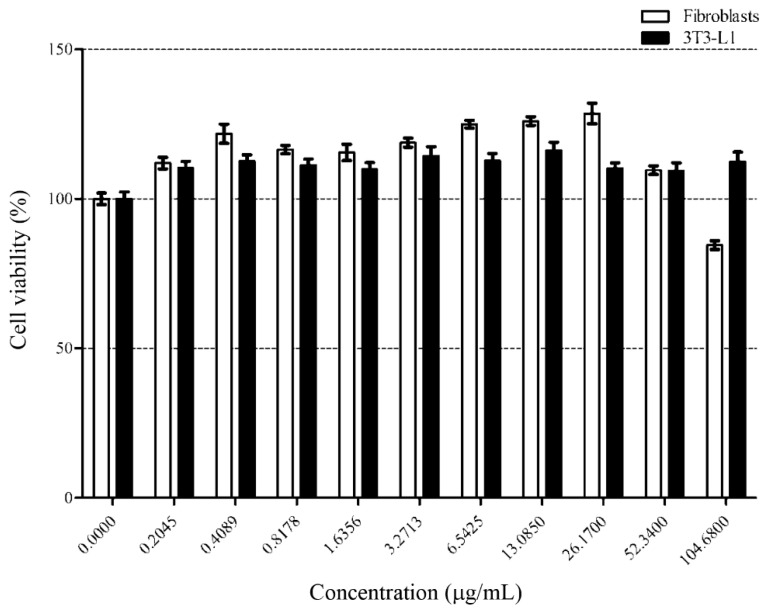
Fibroblast and differentiated adipocyte cells were treated with increasing concentration of loaded liposomes for 24 h. % cell viability was measured by MTT assay. Symbols, (■) and (☐), represent the differentiated 3T3-L1 cells and fibroblast cells, respectively. *y*-axis represents the percentage of cell viability and *x*-axis represents concentrations of the loaded liposome. Data are shown as the mean ± SD from triplicate results.

The safety of using the loaded liposomes is a crucial factor for establishing commercialized products. Therefore, we investigated cell cytotoxicity to evaluate the safety of loaded liposomes using human fibroblasts as normal cell controls and the differentiated 3T3-L1 adipocyte cell line as a lipid storage cell model. Cell viability was measured through an MTT assay and illustrated in Figure 2.

As a result, the loaded liposomes did not significantly affect the viability of either cell lines at concentrations up to 52.34 μg/mL. However, a further increment (104.68 μg/mL) resulted in slight cytotoxic effects on the fibroblast cells. Therefore, we considered the cytotoxicity-related no-observed-adverse-effect level of the loaded liposomes was 52.34 μg/mL for further experiments. Oral delivery of liposomal protein and peptide is the easy and convenient route. The liposome particles made by cholesterol and lecithin were moderately stable (~80% stability measured by particle leakage) in gastric environment (pH 2) at 37 °C at 1 h and stable (~95% stability measured by particle leakage) in pancreatin [30]. These results indicate that our liposome formulations may be suitable as oral delivery particles due to their stable behavior through the oral route. As the potential application of the loaded liposome would be in functional food ingredients, this concentration was used in the determination of lipolysis activity and proteomics.

**Figure 3:**
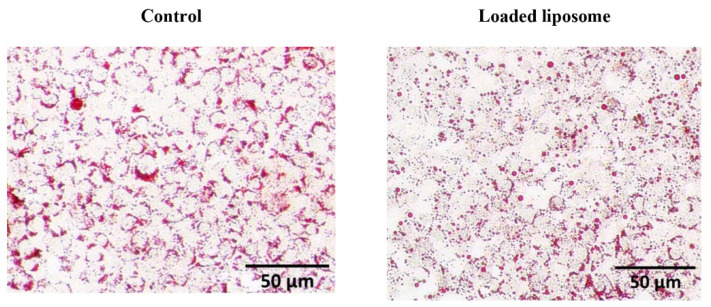
Lipolysis effects of the loaded liposome on the differentiated adipocyte cells. The ORO lipid staining of 3T3-L1 adipocytes was observed using a stereomicroscope at 5× magnification. The cells with no treatment were used as a control (Control).

The lipolysis process is a metabolic process that breaks down TGs to free fatty acid (FA) and glycerol. It controls the energy homeostasis by regulating the breakdown of TGs [31]. Therefore, the effect of 52.34 μg/mL loaded liposome on the TG breakdown in adipocyte cells was investigated through the measurement of glycerol released into the medium culture. In the present study, the loaded liposome significantly increased glycerol release and reduced lipid accumulation. The loaded nanoliposome affected the adipocytes by inducing the TG breakdown, as we observed the release of glycerol at 1.63 ± 0.25-fold greater than that in the control (*p* < 0.01). The intracellular lipid exposed by the loaded nanoliposome was visualized by ORO staining where the lower staining intensity represented the lower lipid accumulation (Figure 3). 

The ORO staining demonstrated lower intracellular lipid accumulation in cells exposed to loaded liposomes compared to the control. The loaded liposome increased glycerol release corresponding to 50% release at 13.085 µg/mL. ORO staining revealed the most pronounced TG clearance at a peak concentration (52.34 µg/mL), with lower staining severity representing lower lipid aggregation (Figure 3). This evidence implied that the loaded nanoliposomes were able to reduce the lipid accumulation as determined by the reduced ORO staining level and the free glycerol level increase. Therefore, we next applied a label-free proteomics approach to study the molecular mechanisms of lipid breakdown activity that could potentially lead to the reduced lipid accumulation in the adipocytes for a better understanding of the loaded liposome-induced lipolytic pathways.

### 3.4. Quantitative Proteomic Analysis

**Figure 4:**
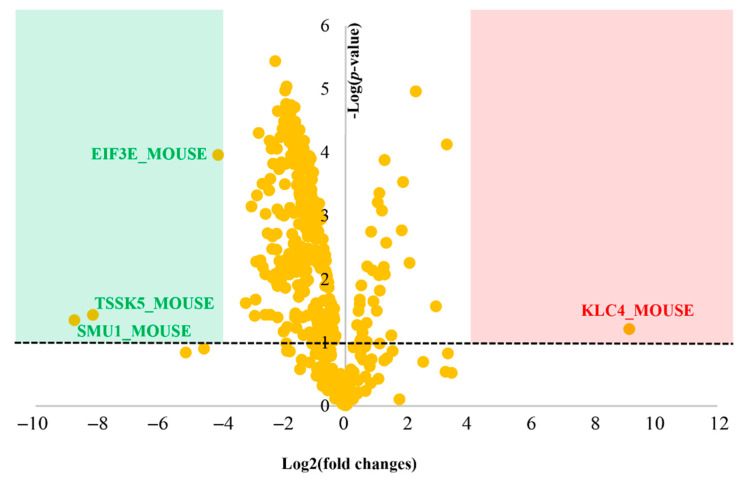
Quantitative proteomic analysis visualized by a volcano plot. The plot shows a negative natural log of the *p*-values plotted against the base2 logs of the change in each protein compared between the loaded liposome and control groups. Statistically significant results (*p* < 0.05) are plotted above the dashed line in the green and red regions. Proteins significantly up- and down-regulated upon the loaded liposome treatment are shown as red and green dots, respectively.

We used a proteomics approach to investigate the signaling pathways that could be potentially affected by the loaded liposomes in the adipocyte cells. The LC-MS/MS analysis revealed a total number of 3425 proteins among the loaded liposome and the control groups. The interpretation of the quantitative proteomics and bioinformatics data showed that 439 proteins were affected by the loaded liposomes as shown in Figure 4. Although we used differentiated adipocytes from mice, this was a widely accepted cell-based model [32]. The raw data from the LC-MS/MS analysis showed a small difference in the total ion count between each LC-MS injection. Therefore, data normalization of the raw dataset was strongly required prior to further analysis. After the log transformation and VSN normalization, pooled intragroup median absolute deviation (PMAD) of the identified proteins among replicates was lower than 0.22 (Control and loaded liposomes *n* = 3 and 3, respectively; Figure S3). In general, a PMAD value of ≤0.3 was accepted as the superior precision dataset [33]. According to the normalized proteomic analysis, the volcano plot of the differential protein expression identifying the most significant protein expression changes is depicted in Figure 4. Each spot represents the protein expression ratio (loaded liposome: control) according to their log_10_ *p*-values. The differentially expressed proteins associated with these spots are listed in the proteomics table (Appendix B).

We identified four significantly different proteins, compared between the loaded liposome and control groups. The global protein expression changes were mostly down-regulated (79.37%; for 350 of 441 proteins). Specifically, three significantly different proteins (*p* < 0.05 and −4 > log_2_ (fold change) > 4) were down-regulated (green region, Figure 4) whereas one was up-regulated (red region, Figure 3). Considering the biological functions of the significantly different proteins, the down-regulated ones were Testis-specific serine/threonine-protein kinase 5 (TSSK5_MOUSE), WD40 repeat-containing protein SMU1 (SMU1_MOUSE), and metabotropic glutamate receptor 7 (GRM7_MOUSE), whereas the up-regulated one was Kinesin light chain 4 (KLC4_MOUSE). The detailed description and function of these proteins are presented in Table 3. 

The biological functions of these proteins were variable, including cell differentiation, intracellular signal transduction, organism development, protein phosphorylation, spermatogenesis, mRNA splicing, cAMP-related G protein inhibition, chemical synapsis-related activities, and the regulation of neuronal death. Notably, the liposome-encapsulated protein hydrolysates affected the 3T3-L1 cells in various biological functions beyond lipolysis.

Although these significant proteins were not directly associated with lipolysis, differentially expressed proteins in lipid biosynthesis and lipolysis could also be identified. Our investigation detected that fatty acid synthase (FAS; FAS_MOUSE), the major actor of lipogenesis, was suppressed more than 5-fold (log_2_ fold change as 2.35) in the loaded liposome group (supplementary data 2). The lipogenesis works via FAS to synthesize the long-chain FA from acetyl-CoA, malonyl-CoA, and NADPH. Hence, FAS downregulation could imply that cellular lipogenesis might be reduced due to the decrease in its abundance and activity. FAS-down regulation, an increased rate of lipolysis, and TG release could lead to a net TG loss on the cellular level. Moreover, another protein that elongates the long-chain fatty acids, protein 5 (ELOV5_MOUSE), was also down-regulated. Elov5, known as PUFA elongase, is a major PPARα-regulated enzyme functioning in monounsaturated and polyunsaturated fatty acid synthesis [34].

## 4. Conclusions

The concordance between the proteomics results and the cellular lipidemic activity could imply that the Lingzhi protein hydrolysate-loaded nano-liposomes induced cellular lipolysis without affecting cell viability. The proteomic study also indicated that loaded liposomes exhibited lipid accumulation with the suppression of FAS and ELOV5. Finally, other proteins including TSSK5, SMU1, GRM7_MOUSE, and KLC4, were identified in the loaded liposome treatment group, associated with various biological mechanisms beyond lipid metabolism. Therefore, the nano-liposomal hydrolysates might serve as novel functional ingredients in the treatment and prevention of obesity.

## Figures and Tables

**Table 1:** The experimental design and experimental outputs of the independent factors for the degree of hydrolysate and yield produced from Lingzhi proteins.

Experiment No.	Independent Factors		Experimental Outputs	
	x_1_; Time (Hour)	x_2_; Enzyme (%)	y_1_; DH (%)	y_2_; Yield (%)
1	3	0.25	28.11 ± 1.03	4.16 ± 0.13
2	3	0.50	29.83 ± 1.30	4.57 ± 0.17
3	3	1.00	29.36 ± 1.28	4.22 ± 0.15
4	6	0.50	33.21 ± 1.03	5.25 ± 0.12
5	6	1.00	32.91 ± 1.37	5.32 ± 0.14
6	6	2.00	32.03 ± 0.76	5.21 ± 0.22
7	9	0.25	33.96 ± 1.14	5.58 ± 0.16
8	9	0.50	33.17 ± 1.29	5.67 ± 0.09
9	9	1.00	34.18 ± 1.12	5.70 ± 0.20
10	6	0.50	33.16 ± 0.58	5.24 ± 0.09
11	6	0.50	32.92 ± 0.32	5.21 ± 0.13

**Table 2:** The experimental design and experimental outputs of the independent factors for the degree of hydrolysate and yield produced from Lingzhi proteins.

Responding	Quadratic Model	*R* ^2^	*p*-Value
y_1_	y_1_ = 33.14 + 2.08x_1_ − 0.497x_2_ − 0.283x_1_x_2_ − 1.53x_1_^2^ − 0.635x_2_^2^	0.96	0.0019
y_2_	y_2_ = 5.293 + 0.726x_1_ − 0.027x_2_ − 0.066x_1_x_2_ − 0.264x_1_^2^ − 0.073x_2_^2^	0.97	0.0010

**Table 3:** The description and functions of the top 4 significant proteins uniquely identified in the liposome-encapsulated hydrolysate treatment group. This information was obtained from the UniProtKB database.

Accession	Protein Name	Biological Process
TSSK5_MOUSE	Testis-specific serine/threonine-protein kinase 5	Cell differentiation, intracellular signal transduction, multicellular organism development, protein phosphorylation, and spermatogenesis
SMU1_MOUSE	WD40 repeat-containing protein SMU1	mRNA splicing, via spliceosome, regulation of alternative mRNA splicing, via spliceosome, and RNA splicing
GRM7_MOUSE	Metabotropic glutamate receptor 7	adenylate cyclase-inhibiting G protein-coupled glutamate receptor signaling pathway, chemical synaptic transmission, and regulation of neuron death
KLC4_MOUSE	Kinesin light chain 4	-

## Data Availability

Not applicable.

## Supplementary Materials

The following are available online at https://www.mdpi.com/article/10.3390/foods10092157/s1, Figure S1: DLS analysis showed size distribution, Figure S2: DLS analysis showed zeta-potential distribution, Figure S3: Intragroup variation analysis.

## Appendix A

**Table A1:** The observed effects due to the hydrolysis variables including digestion time (A) and enzyme usage (B) on the response values for degree of hydrolysis (DH) and %yield.

**1. DH**						
**Model**	39.47	5	7.89	22.9	0.0019	significant
A-Time	4.68	1	4.68	13.56	0.0143	
B-Enzyme	0.769	1	0.769	2.23	0.1955	
AB	0.083	1	0.083	0.2407	0.6445	
A²	5.7	1	5.7	16.54	0.0097	
B²	1.06	1	1.06	3.06	0.1405	
**Residual**	1.72	5	0.3447			
Lack of Fit	1.68	3	0.5585	23.24	0.0415	significant
Pure Error	0.0481	2	0.024			
**Cor Total**	41.19	10				
**Std. Dev.**	0.5871	**R²**			0.9582	
**Mean**	32.08	**Adjusted R²**			0.9163	
**C.V. %**	1.83	**Predicted R²**			0.4581	
		**Adeq Precision**			11.99244	
**2. Yield**						
**Model**	2.87	5	0.5747	30.13	0.001	significant
A-Time	0.5672	1	0.5672	29.74	0.0028	
B-Enzyme	0.0023	1	0.0023	0.1184	0.7448	
AB	0.0046	1	0.0046	0.2399	0.645	
A²	0.1691	1	0.1691	8.87	0.0309	
B²	0.0139	1	0.0139	0.7302	0.4318	
**Residual**	0.0954	5	0.0191			
Lack of Fit	0.0945	3	0.0315	72.69	0.0136	significant
Pure Error	0.0009	2	0.0004			
**Cor Total**	2.97	10				
**Std. Dev.**	0.1381	**R²**			0.9679	
**Mean**	5.1	**Adjusted R²**			0.9358	
**C.V. %**	2.71	**Predicted R²**			−0.5277	
		**Adeq Precision**			14.0722	

## Appendix B

**Table A2:** Differentially expressed proteins list.

Protein Name	*p*-Value	Log2 Intensity						
		Control01	Control02	Control03	Liposome01	Liposome02	Liposome03	log(*p*-Value)
HNRH1_MOUSE	3.65 × 10^−6^	16.58	16.46	16.41	14.17	14.23	14.26	5.44
VIME_MOUSE	9.17 × 10^−6^	19.58	19.48	19.52	17.64	17.55	17.70	5.04
TBA8_MOUSE	1.06 × 10^−5^	19.94	19.82	19.70	17.87	17.87	17.90	4.98
HIG1A_MOUSE	1.72 × 10^−5^	13.17	13.12	12.98	11.23	11.27	11.04	4.76
UBA1_MOUSE	1.80 × 10^−5^	17.44	17.45	17.35	15.74	15.70	15.54	4.75
IF4A1_MOUSE	1.96 × 10^−5^	16.22	16.32	16.30	14.65	14.57	14.71	4.71
KPYM_MOUSE	2.20 × 10^−5^	20.80	21.00	20.79	18.90	19.10	18.83	4.66
PSMD2_MOUSE	2.24 × 10^−5^	15.41	15.44	15.06	12.99	13.21	13.17	4.65
TBA4A_MOUSE	2.42 × 10^−5^	20.35	20.20	20.09	18.36	18.42	18.53	4.62
ATPA_MOUSE	3.24 × 10^−5^	18.45	18.15	18.20	16.13	16.46	16.29	4.49
IPO5_MOUSE	3.30 × 10^−5^	15.47	15.28	15.53	13.84	13.79	13.80	4.48
RAN_MOUSE	3.41 × 10^−5^	18.54	18.40	18.76	16.53	16.64	16.78	4.47
RANT_MOUSE	3.41 × 10^−5^	18.54	18.40	18.76	16.53	16.64	16.78	4.47
RGL2_MOUSE	3.44 × 10^−5^	15.83	15.62	15.75	13.64	13.99	13.91	4.46
MYH9_MOUSE	3.81 × 10^−5^	17.63	17.64	17.86	15.73	15.94	16.04	4.42
ADT2_MOUSE	4.25 × 10^−5^	18.79	19.10	18.81	16.78	17.10	16.74	4.37
ADT1_MOUSE	4.37 × 10^−5^	18.12	18.30	18.16	16.21	16.60	16.37	4.36
RHOA_MOUSE	4.43 × 10^−5^	15.32	15.23	15.38	13.93	13.79	13.77	4.35
GLRX3_MOUSE	4.53 × 10^−5^	16.10	16.22	15.99	14.55	14.36	14.56	4.34
FLNA_MOUSE	4.90 × 10^−5^	17.85	18.20	18.00	16.40	16.38	16.38	4.31
SYSC_MOUSE	4.95 × 10^−5^	12.81	13.18	12.30	9.98	10.00	9.94	4.31
SDHA_MOUSE	5.34 × 10^−5^	15.09	14.78	15.07	13.39	13.18	13.18	4.27
PRS6B_MOUSE	5.61 × 10^−5^	15.29	15.71	15.39	13.70	13.69	13.52	4.25
CAN2_MOUSE	5.62 × 10^−5^	12.78	13.08	12.52	10.78	10.59	10.80	4.25
ATAD1_MOUSE	5.75 × 10^−5^	16.37	16.22	15.98	14.27	14.20	13.83	4.24
RS2_MOUSE	6.36 × 10^−5^	16.51	16.43	16.18	14.73	14.83	14.62	4.20
NONO_MOUSE	6.55 × 10^−5^	15.03	15.20	15.10	13.09	12.32	12.58	4.18
PGM1_MOUSE	6.58 × 10^−5^	15.08	15.23	15.20	13.77	13.89	13.85	4.18
EF2_MOUSE	6.83 × 10^−5^	19.20	19.04	19.08	17.60	17.80	17.57	4.17
FINC_MOUSE	6.87 × 10^−5^	16.44	16.08	16.53	14.48	14.55	14.63	4.16
K22O_MOUSE	7.50 × 10^−5^	15.65	15.76	14.98	18.79	19.12	18.30	4.13
TCPB_MOUSE	8.00 × 10^−5^	15.64	15.75	15.50	14.27	14.01	14.12	4.10
SYVC_MOUSE	8.03 × 10^−5^	13.57	14.21	13.62	11.53	11.61	11.32	4.10
PRDX2_MOUSE	8.32 × 10^−5^	16.59	16.25	16.50	14.71	14.65	14.95	4.08
SERPH_MOUSE	8.67 × 10^−5^	17.83	18.03	17.99	16.55	16.70	16.54	4.06
APT_MOUSE	8.76 × 10^−5^	13.68	13.87	14.07	11.55	12.03	11.42	4.06
MYH10_MOUSE	8.77 × 10^−5^	14.63	14.66	14.52	11.93	12.05	12.69	4.06
HS90A_MOUSE	9.32 × 10^−5^	19.67	19.82	19.74	18.19	18.48	18.32	4.03
EIF3E_MOUSE	0.000109	12.02	13.07	13.27	8.21	9.18	8.62	3.96
COF1_MOUSE	0.000111	20.53	20.48	20.50	19.26	19.40	19.20	3.95
TBB5_MOUSE	0.000112	18.99	18.79	18.92	17.67	17.69	17.69	3.95
TBA1C_MOUSE	0.000112	21.61	21.08	21.35	19.63	19.46	19.68	3.95
RL6_MOUSE	0.000116	14.96	14.92	14.97	13.88	13.76	13.76	3.94
TBA1B_MOUSE	0.000118	21.62	21.10	21.37	19.64	19.50	19.72	3.93
TBB4B_MOUSE	0.000118	18.99	18.77	18.91	17.62	17.68	17.68	3.93
TBA1A_MOUSE	0.000122	21.32	20.84	21.12	19.51	19.37	19.53	3.91
TERA_MOUSE	0.000125	19.24	19.22	19.26	18.07	18.18	18.12	3.90
PP1B_MOUSE	0.000129	16.74	16.73	16.63	15.62	15.53	15.55	3.89
K1C15_MOUSE	0.000132	15.09	14.81	14.93	16.24	16.17	16.23	3.88
K1C17_MOUSE	0.000132	15.09	14.81	14.93	16.24	16.17	16.23	3.88
ATPB_MOUSE	0.000137	19.50	19.44	19.37	18.08	18.18	18.33	3.86
HS90B_MOUSE	0.000139	20.21	20.04	20.30	18.75	18.97	18.77	3.86
IF4G1_MOUSE	0.000144	13.62	13.50	13.27	11.78	11.10	11.33	3.84
CPSF5_MOUSE	0.000148	14.38	14.59	14.62	13.23	13.35	13.31	3.83
MYADM_MOUSE	0.000151	13.23	13.16	13.01	11.29	10.72	10.41	3.82
S35E3_MOUSE	0.000152	12.63	13.06	12.52	11.02	11.11	11.07	3.82
PUR6_MOUSE	0.000153	18.17	17.71	17.96	15.97	16.31	15.75	3.82
ANXA2_MOUSE	0.000156	20.59	20.84	20.61	19.12	19.44	19.25	3.81
RS3_MOUSE	0.000157	18.82	18.55	18.71	16.61	16.87	16.09	3.81
PDIA1_MOUSE	0.000158	20.30	20.30	20.23	18.96	19.21	19.09	3.80
HNRPF_MOUSE	0.00016	16.04	15.88	16.21	14.59	14.28	14.65	3.79
FLNB_MOUSE	0.000165	16.61	16.89	16.76	15.53	15.25	15.21	3.78
LDHA_MOUSE	0.000169	19.05	19.17	19.18	17.69	18.00	17.67	3.77
RL11_MOUSE	0.000175	17.54	17.32	17.35	15.90	16.21	15.90	3.76
FLNC_MOUSE	0.000184	16.60	16.87	16.57	15.41	15.23	15.42	3.74
MVP_MOUSE	0.000184	15.70	15.74	15.43	13.92	13.14	13.40	3.73
HSP7C_MOUSE	0.000191	19.22	19.02	18.99	17.98	17.88	17.86	3.72
ALDOA_MOUSE	0.000206	18.40	18.48	18.51	17.48	17.40	17.39	3.69
PP1G_MOUSE	0.000222	16.26	16.32	16.27	15.30	15.05	15.16	3.65
PP1A_MOUSE	0.000222	16.26	16.32	16.27	15.30	15.05	15.16	3.65
PSMD5_MOUSE	0.000233	15.59	15.92	15.73	14.00	14.46	14.11	3.63
PROF1_MOUSE	0.000243	20.22	19.93	19.78	18.46	18.31	18.66	3.61
XPO2_MOUSE	0.000262	12.68	12.49	12.25	10.12	10.52	9.52	3.58
LMNA_MOUSE	0.00027	16.59	16.69	16.44	15.47	15.54	15.47	3.57
TDRD1_MOUSE	0.000295	13.59	13.62	13.36	15.73	15.45	14.98	3.53
TIF1B_MOUSE	0.000299	16.56	16.31	16.41	15.27	15.25	14.94	3.52
RPN2_MOUSE	0.000314	15.97	16.00	16.01	13.80	12.59	13.57	3.50
HNRPU_MOUSE	0.000319	17.21	17.24	17.36	14.85	15.72	15.38	3.50
RL18A_MOUSE	0.000329	14.71	14.44	14.93	13.18	12.89	13.32	3.48
ERO1A_MOUSE	0.000386	15.67	15.38	15.62	14.37	14.48	14.54	3.41
TCPZ_MOUSE	0.000399	16.61	16.71	16.55	14.43	14.60	13.47	3.40
SCMC1_MOUSE	0.00041	14.28	14.38	14.72	13.11	13.18	12.81	3.39
TM183_MOUSE	0.00044	15.32	15.38	15.66	16.48	16.56	16.60	3.36
HSP74_MOUSE	0.000448	15.18	15.18	15.29	13.44	13.68	14.09	3.35
SMD1_MOUSE	0.000461	16.87	16.91	17.00	15.65	15.81	16.02	3.34
CH10_MOUSE	0.000468	16.69	16.69	16.73	15.41	15.80	15.57	3.33
UGDH_MOUSE	0.000477	14.07	14.57	13.84	11.66	10.55	11.72	3.32
CAP1_MOUSE	0.000498	16.30	16.31	16.47	14.58	15.23	14.87	3.30
ENPL_MOUSE	0.000579	18.40	18.37	18.66	17.38	17.53	17.48	3.24
K1C10_MOUSE	0.000616	19.19	18.83	19.03	19.97	20.10	20.12	3.21
2AAB_MOUSE	0.000622	16.25	16.35	16.31	15.06	15.08	15.45	3.21
IF5A1_MOUSE	0.000629	19.07	18.46	18.51	17.27	17.30	17.22	3.20
GDIR1_MOUSE	0.000645	18.34	18.29	18.31	17.37	17.53	17.51	3.19
AATM_MOUSE	0.000648	17.71	17.39	17.46	16.14	16.46	16.46	3.19
RTN4_MOUSE	0.000686	18.65	18.56	18.47	17.45	17.67	17.70	3.16
IMA3_MOUSE	0.000702	14.57	14.31	14.34	13.11	12.85	13.40	3.15
VINC_MOUSE	0.000715	15.15	15.11	15.34	14.37	14.18	14.12	3.15
IPO9_MOUSE	0.000716	13.49	13.79	13.48	9.63	10.70	11.33	3.15
PTBP1_MOUSE	0.000751	16.80	16.77	16.35	15.15	15.03	14.37	3.12
TCPE_MOUSE	0.000793	15.29	15.48	15.26	12.43	13.52	13.49	3.10
ROA2_MOUSE	0.000823	17.53	17.74	17.68	16.65	16.82	16.47	3.08
DYH17_MOUSE	0.000832	15.79	15.55	16.03	16.81	17.02	17.08	3.08
CLH1_MOUSE	0.000871	16.53	16.87	16.48	15.33	15.18	14.61	3.06
PDIA6_MOUSE	0.0009	17.40	17.61	17.45	16.51	16.69	16.65	3.05
TCPH_MOUSE	0.000902	15.71	15.81	15.83	14.01	14.55	14.69	3.04
TSN_MOUSE	0.000904	13.78	14.16	13.83	12.71	12.19	11.89	3.04
BIP_MOUSE	0.000924	19.73	19.42	19.53	18.43	18.63	18.67	3.03
ARP2_MOUSE	0.000936	13.07	13.22	12.74	11.18	9.70	10.44	3.03
AP2B1_MOUSE	0.000944	13.83	13.51	14.01	12.35	11.43	11.32	3.02
DDX3X_MOUSE	0.001	14.39	14.57	14.65	13.03	11.91	12.73	3.00
ALBU_MOUSE	0.001032	17.91	17.81	17.73	16.88	17.06	17.03	2.99
RSSA_MOUSE	0.001081	18.25	18.16	18.23	17.10	17.49	17.15	2.97
2AAA_MOUSE	0.001131	16.75	16.82	16.86	15.95	16.02	16.14	2.95
CATB_MOUSE	0.001131	15.43	15.32	15.81	14.43	14.49	14.20	2.95
NNRE_MOUSE	0.001157	11.06	10.70	11.31	9.55	9.59	9.94	2.94
TBB2B_MOUSE	0.001168	18.07	17.91	18.20	17.23	17.25	17.22	2.93
TBB2A_MOUSE	0.001168	18.07	17.91	18.20	17.23	17.25	17.22	2.93
ACTN4_MOUSE	0.001328	17.08	16.94	17.30	16.31	16.09	16.14	2.88
ERO1B_MOUSE	0.001349	14.30	13.93	14.27	12.28	12.63	13.09	2.87
6PGL_MOUSE	0.001531	15.37	15.81	15.86	14.54	14.71	14.65	2.81
RAP1B_MOUSE	0.001542	15.48	15.07	15.33	14.30	13.64	14.04	2.81
RAP1A_MOUSE	0.001542	15.48	15.07	15.33	14.30	13.64	14.04	2.81
ANXA6_MOUSE	0.001692	16.80	16.23	16.23	15.02	15.16	15.38	2.77
S10AB_MOUSE	0.001703	13.22	13.31	14.28	15.26	15.56	15.45	2.77
PPIB_MOUSE	0.001762	15.44	15.65	15.39	14.45	14.78	14.62	2.75
H14_MOUSE	0.00179	17.02	16.91	17.20	17.87	18.00	17.75	2.75
API5_MOUSE	0.001801	14.71	14.07	14.21	12.94	13.28	13.09	2.74
MDGA1_MOUSE	0.001891	19.07	18.32	17.69	16.43	15.32	15.80	2.72
SRPRA_MOUSE	0.001922	13.29	12.49	12.48	10.53	11.16	9.90	2.72
FKB1A_MOUSE	0.001939	13.86	13.87	14.02	13.22	12.72	12.76	2.71
CDC42_MOUSE	0.001946	17.24	17.13	17.09	14.77	15.74	15.80	2.71
FAS_MOUSE	0.002116	13.77	13.67	14.08	10.58	12.16	11.74	2.67
PLAK_MOUSE	0.002129	14.54	14.51	14.25	13.56	13.25	12.85	2.67
MDHM_MOUSE	0.002326	20.02	20.21	20.00	19.18	19.44	19.34	2.63
ANXA1_MOUSE	0.002341	16.96	16.78	17.14	15.77	16.14	16.13	2.63
PLCB1_MOUSE	0.002343	17.38	17.61	17.54	16.79	16.68	16.88	2.63
KAP0_MOUSE	0.002469	13.88	14.01	13.80	12.76	11.66	12.46	2.61
RAGP1_MOUSE	0.00262	14.51	14.81	14.25	13.50	12.75	12.53	2.58
BIRC2_MOUSE	0.002678	16.61	15.81	16.19	17.24	17.66	17.66	2.57
MOES_MOUSE	0.002759	15.45	15.26	15.56	13.43	14.44	13.37	2.56
KAD2_MOUSE	0.003069	13.79	14.07	13.67	12.41	12.90	11.92	2.51
R51A1_MOUSE	0.0031	12.95	13.00	13.56	11.02	12.05	11.58	2.51
RS7_MOUSE	0.003174	17.66	16.56	16.77	15.44	15.54	15.31	2.50
RLA0_MOUSE	0.0033	18.50	18.44	17.93	16.74	17.30	17.22	2.48
K2C1_MOUSE	0.003341	18.33	18.24	18.33	17.56	17.78	17.46	2.48
SF3B1_MOUSE	0.003346	12.56	13.20	12.99	10.58	9.66	11.43	2.48
SON_MOUSE	0.003363	15.63	16.38	16.05	14.59	15.01	14.28	2.47
USO1_MOUSE	0.003429	12.17	12.17	12.45	9.20	10.95	9.98	2.46
IMB1_MOUSE	0.003496	17.29	17.51	16.97	16.38	16.41	16.06	2.46
S10A6_MOUSE	0.003578	20.89	21.05	20.66	19.87	19.80	19.06	2.45
PPIA_MOUSE	0.003862	20.57	20.18	20.41	19.71	19.67	19.62	2.41
RL14_MOUSE	0.003872	16.09	15.94	15.68	14.82	14.68	15.23	2.41
RS8_MOUSE	0.003989	15.56	15.41	15.45	13.37	14.24	14.45	2.40
PRDX1_MOUSE	0.003992	18.09	18.49	18.10	17.08	17.53	16.93	2.40
MBB1A_MOUSE	0.00401	13.93	13.88	13.57	12.52	12.26	11.16	2.40
EF1A1_MOUSE	0.004196	18.52	18.47	18.60	17.73	18.02	17.90	2.38
SEPT2_MOUSE	0.004277	15.38	14.98	15.32	14.00	13.59	14.44	2.37
ARF1_MOUSE	0.004524	16.52	16.29	16.32	13.92	15.25	14.93	2.34
ADK_MOUSE	0.004525	14.30	14.42	14.35	12.92	13.04	13.73	2.34
ATPK_MOUSE	0.004911	16.16	15.57	14.97	13.98	14.08	14.28	2.31
VDAC3_MOUSE	0.004948	15.00	15.20	14.34	13.66	13.76	13.72	2.31
RACK1_MOUSE	0.005006	15.85	16.09	16.52	14.62	13.34	14.72	2.30
VATL_MOUSE	0.005044	14.01	14.17	13.35	12.02	11.46	9.83	2.30
PRDX4_MOUSE	0.005054	17.40	17.89	17.57	16.65	16.98	16.66	2.30
NUCL_MOUSE	0.005137	19.18	18.99	18.89	18.37	18.33	18.48	2.29
SRP68_MOUSE	0.00519	13.00	13.38	13.26	11.72	10.03	11.51	2.28
4F2_MOUSE	0.005313	14.17	13.88	13.85	12.39	13.22	12.15	2.27
RIC8B_MOUSE	0.005318	14.21	14.14	14.62	10.00	12.37	11.96	2.27
K1C14_MOUSE	0.005548	14.01	14.36	12.72	15.38	16.00	15.93	2.26
UBA6_MOUSE	0.005564	11.49	11.66	10.14	9.14	9.39	9.00	2.25
RL7A_MOUSE	0.005709	15.36	15.63	14.99	14.33	14.52	14.49	2.24
SAP_MOUSE	0.006308	18.39	18.62	18.60	19.01	19.41	19.32	2.20
MYL6_MOUSE	0.006405	15.38	15.40	16.33	16.91	16.93	17.00	2.19
GTR1_MOUSE	0.006448	14.20	13.70	14.27	10.09	12.49	11.59	2.19
CSN4_MOUSE	0.006496	11.88	11.84	12.08	9.60	10.34	11.04	2.19
UGGG1_MOUSE	0.006589	12.08	13.06	13.32	10.28	11.08	11.42	2.18
TCPD_MOUSE	0.006742	14.59	14.85	14.80	14.23	13.67	13.94	2.17
IMA7_MOUSE	0.007097	12.27	12.58	12.13	9.33	11.25	10.13	2.15
K1C13_MOUSE	0.007257	17.30	16.66	16.85	17.75	17.99	17.70	2.14
VDAC2_MOUSE	0.007331	15.12	15.16	15.24	14.16	13.27	14.32	2.13
GRP75_MOUSE	0.007397	18.49	18.38	18.09	17.63	17.72	17.69	2.13
HEAT3_MOUSE	0.007572	12.46	11.35	12.59	10.59	10.68	10.72	2.12
SMRC1_MOUSE	0.007688	11.23	11.33	11.38	9.91	10.38	8.91	2.11
CDK1_MOUSE	0.007805	9.17	10.81	10.28	7.99	8.33	8.49	2.11
AL7A1_MOUSE	0.008283	14.00	13.86	13.60	10.32	12.37	12.01	2.08
PGAM1_MOUSE	0.008302	20.10	19.97	20.04	19.21	19.51	19.57	2.08
ACON_MOUSE	0.008378	14.44	14.12	13.91	11.95	10.17	12.61	2.08
UB2D2_MOUSE	0.008403	13.84	14.98	14.95	12.17	12.82	13.37	2.08
UB2D3_MOUSE	0.008403	13.84	14.98	14.95	12.17	12.82	13.37	2.08
K1C42_MOUSE	0.008429	15.76	16.13	15.29	16.58	17.00	17.44	2.07
K22E_MOUSE	0.008676	17.43	17.32	17.02	17.85	18.75	18.42	2.06
DPY30_MOUSE	0.009099	14.38	14.41	14.24	13.73	13.58	13.94	2.04
CAND1_MOUSE	0.009439	13.46	14.71	15.00	12.52	12.57	11.02	2.03
PYRG1_MOUSE	0.00988	13.91	14.16	13.52	11.86	10.76	12.78	2.01
AT5G1_MOUSE	0.010402	14.30	14.61	14.59	13.56	13.68	12.63	1.98
AT5G3_MOUSE	0.010402	14.30	14.61	14.59	13.56	13.68	12.63	1.98
AT5G2_MOUSE	0.010402	14.30	14.61	14.59	13.56	13.68	12.63	1.98
TLN1_MOUSE	0.010823	12.88	13.67	13.19	11.53	12.54	11.78	1.97
HNRPL_MOUSE	0.011041	14.09	14.70	14.00	12.95	13.54	12.50	1.96
CKAP4_MOUSE	0.011183	15.39	15.64	15.70	14.98	14.96	15.14	1.95
RPN1_MOUSE	0.011709	13.95	13.88	14.23	12.28	12.69	10.48	1.93
PFKAL_MOUSE	0.01242	14.66	15.33	14.61	13.56	12.54	13.92	1.91
H13_MOUSE	0.01257	17.64	17.48	17.53	18.10	18.20	17.90	1.90
CSN7A_MOUSE	0.012775	13.73	13.81	13.95	13.46	13.15	12.94	1.89
LKHA4_MOUSE	0.012846	12.57	12.89	11.55	9.83	11.25	9.36	1.89
RLA2_MOUSE	0.013592	19.15	19.23	19.07	18.41	18.77	18.66	1.87
EIF3F_MOUSE	0.01375	13.49	13.62	13.54	11.75	12.62	10.47	1.86
C1QBP_MOUSE	0.01467	15.43	15.09	15.42	14.74	13.61	13.23	1.83
TAGL2_MOUSE	0.0152	14.60	14.88	15.73	16.05	16.15	16.36	1.82
DESP_MOUSE	0.015762	13.61	12.78	12.85	11.29	12.42	11.54	1.80
OTUB1_MOUSE	0.019088	14.60	14.23	13.91	12.65	11.96	13.63	1.72
CALX_MOUSE	0.020499	16.20	15.75	16.00	15.51	15.44	15.45	1.69
H12_MOUSE	0.021046	16.68	16.42	16.57	16.88	17.14	17.05	1.68
ATPG_MOUSE	0.02109	14.11	13.07	13.84	10.70	12.56	9.07	1.68
LAMP2_MOUSE	0.021862	16.01	15.97	15.44	14.86	15.13	15.31	1.66
PSA5_MOUSE	0.022314	15.01	15.16	15.13	14.48	14.81	14.20	1.65
TRY2_MOUSE	0.022394	23.38	23.70	23.06	24.10	24.78	23.98	1.65
TCPA_MOUSE	0.023869	14.28	14.09	14.44	8.73	12.79	11.64	1.62
RL12_MOUSE	0.024021	15.04	14.26	14.99	14.18	13.84	13.44	1.62
BASP1_MOUSE	0.024344	14.75	14.62	14.68	14.39	14.03	14.27	1.61
K1C19_MOUSE	0.024816	20.16	20.03	20.09	20.34	20.63	20.70	1.61
EF1D_MOUSE	0.026795	15.14	15.40	15.53	13.81	14.69	14.81	1.57
K2C4_MOUSE	0.026904	16.58	16.50	16.10	17.07	20.58	20.30	1.57
PRDX5_MOUSE	0.028629	15.67	14.57	15.15	14.44	14.17	13.68	1.54
THIO_MOUSE	0.029175	17.68	17.83	17.73	17.41	17.43	17.31	1.53
YBOX2_MOUSE	0.029181	16.07	15.87	16.09	15.42	15.41	15.77	1.53
YBOX3_MOUSE	0.029181	16.07	15.87	16.09	15.42	15.41	15.77	1.53
TOM20_MOUSE	0.030869	13.51	12.89	13.21	12.85	12.20	11.86	1.51
CH60_MOUSE	0.031589	18.43	18.23	18.18	17.81	17.99	17.84	1.50
CD63_MOUSE	0.03168	13.65	13.30	13.68	13.86	14.66	15.15	1.50
K2C8_MOUSE	0.03168	17.18	16.87	17.34	16.74	16.58	16.62	1.50
TPIS_MOUSE	0.031746	18.13	18.18	18.42	18.56	18.80	18.66	1.50
SODC_MOUSE	0.033924	16.50	16.81	17.01	16.47	15.51	15.88	1.47
SYAC_MOUSE	0.034719	14.61	14.49	14.41	14.20	12.04	12.45	1.46
SMU1_MOUSE	0.036374	12.11	11.53	11.24	0.00	10.41	0.00	1.44
C1TM_MOUSE	0.036422	12.82	13.00	13.37	11.36	8.63	11.91	1.44
ARF4_MOUSE	0.036656	16.11	15.77	15.77	11.12	14.59	14.17	1.44
ANXA5_MOUSE	0.036715	19.32	19.28	19.18	18.85	18.98	18.45	1.44
PARK7_MOUSE	0.037258	13.64	14.02	13.39	12.77	13.09	11.55	1.43
P4HA1_MOUSE	0.037299	14.18	14.14	14.57	13.57	14.02	13.65	1.43
THIKA_MOUSE	0.037977	13.61	13.17	13.46	12.23	8.06	11.17	1.42
MTAP_MOUSE	0.038542	15.04	14.85	14.92	14.72	14.39	14.51	1.41
THIC_MOUSE	0.038635	10.87	11.76	11.13	10.35	10.69	10.57	1.41
LAP2B_MOUSE	0.039043	14.57	14.75	14.52	14.24	14.14	14.35	1.41
NU155_MOUSE	0.039897	12.47	11.90	10.75	10.88	10.26	9.88	1.40
TKT_MOUSE	0.039985	13.72	14.07	13.84	12.88	10.86	13.00	1.40
CO1A1_MOUSE	0.040121	13.56	13.79	12.40	11.31	12.66	11.63	1.40
CAPZB_MOUSE	0.040224	14.17	14.21	13.87	10.09	13.29	12.32	1.40
VDAC1_MOUSE	0.042968	17.38	16.30	17.24	16.26	16.19	15.97	1.37
NP1L1_MOUSE	0.043459	16.89	17.08	17.00	16.56	16.77	16.57	1.36
TSSK5_MOUSE	0.044033	16.69	16.56	16.64	0.00	11.28	12.35	1.36
PEBP1_MOUSE	0.044407	16.86	16.87	17.32	16.39	16.63	16.60	1.35
H4_MOUSE	0.044792	18.83	19.42	18.62	17.86	18.65	17.62	1.35
NPM_MOUSE	0.049164	16.09	15.74	16.14	16.16	16.87	17.01	1.31
EF1B_MOUSE	0.04968	16.12	16.36	16.28	15.74	15.48	16.08	1.30
PSD13_MOUSE	0.051249	11.93	11.08	11.28	10.38	11.13	10.10	1.29
PAIRB_MOUSE	0.05299	15.41	15.12	15.11	15.45	15.65	15.68	1.28
PLEC_MOUSE	0.05689	13.93	14.45	14.22	13.50	12.90	10.57	1.24
H2B1K_MOUSE	0.058515	12.26	12.50	13.57	10.16	12.41	11.01	1.23
H2B1C_MOUSE	0.058515	12.26	12.50	13.57	10.16	12.41	11.01	1.23
H2B1H_MOUSE	0.058515	12.26	12.50	13.57	10.16	12.41	11.01	1.23
H2B1F_MOUSE	0.058515	12.26	12.50	13.57	10.16	12.41	11.01	1.23
H2B3B_MOUSE	0.058515	12.26	12.50	13.57	10.16	12.41	11.01	1.23
H2B3A_MOUSE	0.058515	12.26	12.50	13.57	10.16	12.41	11.01	1.23
H2B1P_MOUSE	0.058515	12.26	12.50	13.57	10.16	12.41	11.01	1.23
H2B2B_MOUSE	0.058515	12.26	12.50	13.57	10.16	12.41	11.01	1.23
H2B2E_MOUSE	0.058515	12.26	12.50	13.57	10.16	12.41	11.01	1.23
H2B1B_MOUSE	0.058515	12.26	12.50	13.57	10.16	12.41	11.01	1.23
H2B1M_MOUSE	0.058515	12.26	12.50	13.57	10.16	12.41	11.01	1.23
HNRPK_MOUSE	0.060011	18.52	18.54	18.47	17.59	18.44	17.70	1.22
KLC4_MOUSE	0.060749	12.56	0.00	14.57	17.84	17.96	18.79	1.22
1433Z_MOUSE	0.061261	18.97	19.31	19.48	18.83	18.90	18.83	1.21
GSHR_MOUSE	0.067255	11.26	11.97	11.15	11.44	8.39	8.57	1.17
RS23_MOUSE	0.067883	14.28	13.20	14.33	13.23	12.76	13.34	1.17
IF4B_MOUSE	0.069049	14.02	14.03	13.57	13.24	13.33	11.59	1.16
CALM3_MOUSE	0.069229	18.81	18.84	18.56	18.90	19.25	19.72	1.16
CALM2_MOUSE	0.069229	18.81	18.84	18.56	18.90	19.25	19.72	1.16
CALM1_MOUSE	0.069229	18.81	18.84	18.56	18.90	19.25	19.72	1.16
ATP5J_MOUSE	0.072899	13.97	14.29	14.26	13.62	13.68	14.03	1.14
COPG1_MOUSE	0.074631	14.63	14.90	14.06	12.58	14.17	13.74	1.13
CPNE1_MOUSE	0.07629	9.39	10.50	11.33	9.91	8.84	8.39	1.12
RAB1B_MOUSE	0.077103	15.28	14.29	14.56	13.56	14.43	13.86	1.11
RAB1A_MOUSE	0.077103	15.28	14.29	14.56	13.56	14.43	13.86	1.11
PCNA_MOUSE	0.077431	14.30	12.74	13.33	12.28	11.76	13.01	1.11
RBBP7_MOUSE	0.077454	13.35	15.62	15.59	15.96	16.83	16.23	1.11
RBBP4_MOUSE	0.077454	13.35	15.62	15.59	15.96	16.83	16.23	1.11
H2AV_MOUSE	0.07878	18.16	18.27	18.00	17.72	17.79	17.97	1.10
H2AZ_MOUSE	0.07878	18.16	18.27	18.00	17.72	17.79	17.97	1.10
AN32A_MOUSE	0.09057	19.92	19.71	19.91	20.11	20.81	20.08	1.04
K2C5_MOUSE	0.090801	18.63	18.91	18.73	17.67	18.00	18.75	1.04
TEBP_MOUSE	0.092832	14.31	14.27	14.67	14.25	13.87	14.03	1.03
AN32B_MOUSE	0.094555	19.89	19.70	19.87	20.08	20.79	20.05	1.02
G3P_MOUSE	0.097292	19.35	19.40	19.66	17.89	19.16	19.09	1.01
LRC59_MOUSE	0.097584	14.27	15.08	14.15	14.70	15.56	15.31	1.01
XPO1_MOUSE	0.099533	13.56	13.24	13.89	13.07	13.38	12.77	1.00
UBE2N_MOUSE	0.100923	15.22	14.93	14.57	14.29	13.85	14.79	1.00
RS3A_MOUSE	0.103532	16.06	15.61	15.98	11.72	15.25	14.96	0.98
CNBP_MOUSE	0.103689	12.35	14.37	14.12	15.04	14.54	14.59	0.98
PHB2_MOUSE	0.104427	14.67	14.13	13.97	13.95	13.60	12.01	0.98
SC61B_MOUSE	0.106335	13.45	13.48	12.63	13.93	14.12	13.38	0.97
P5CS_MOUSE	0.108547	9.54	10.84	10.65	9.90	9.66	8.91	0.96
LBH_MOUSE	0.116272	14.11	13.63	12.91	13.96	14.14	14.49	0.93
ML12B_MOUSE	0.118359	17.10	16.86	16.90	17.04	17.58	17.28	0.93
ENOA_MOUSE	0.119211	19.91	19.66	19.75	19.97	20.15	19.97	0.92
RTRAF_MOUSE	0.120351	9.09	10.08	9.42	9.90	10.19	10.06	0.92
PLBL2_MOUSE	0.125355	10.46	9.89	10.73	9.65	0.00	7.75	0.90
K2C6B_MOUSE	0.134139	18.83	19.04	18.86	17.95	18.42	18.93	0.87
K2C6A_MOUSE	0.134139	18.83	19.04	18.86	17.95	18.42	18.93	0.87
K2C75_MOUSE	0.134139	18.83	19.04	18.86	17.95	18.42	18.93	0.87
RINI_MOUSE	0.135641	10.54	13.30	12.66	8.46	11.63	10.75	0.87
TOM70_MOUSE	0.136482	8.39	10.68	11.45	12.25	12.00	10.84	0.86
NDKA_MOUSE	0.137404	15.14	15.82	16.17	16.20	16.09	16.32	0.86
K1C12_MOUSE	0.138179	14.33	12.40	13.09	11.04	9.90	13.51	0.86
GRM7_MOUSE	0.144146	11.24	12.54	12.50	9.36	11.47	0.00	0.84
RL40_MOUSE	0.147393	19.31	18.88	18.98	18.04	18.14	19.20	0.83
RS27A_MOUSE	0.147393	19.31	18.88	18.98	18.04	18.14	19.20	0.83
UBC_MOUSE	0.147393	19.31	18.88	18.98	18.04	18.14	19.20	0.83
UBB_MOUSE	0.147393	19.31	18.88	18.98	18.04	18.14	19.20	0.83
M21_MPV15	0.148508	8.24	15.22	15.12	16.19	16.17	16.15	0.83
NAA15_MOUSE	0.150828	13.48	13.36	12.60	13.06	12.46	11.03	0.82
RS14_MOUSE	0.151861	14.26	15.89	16.28	14.67	14.50	14.57	0.82
PDIA3_MOUSE	0.153504	15.84	16.03	15.35	15.16	15.70	14.97	0.81
EF1G_MOUSE	0.156411	13.27	13.92	13.27	13.39	13.03	12.04	0.81
LEG1_MOUSE	0.1623	18.70	18.71	18.85	18.59	19.60	19.56	0.79
PGRC1_MOUSE	0.163353	14.32	14.37	13.93	14.08	13.96	13.18	0.79
ECI1_MOUSE	0.163424	11.09	12.41	11.78	11.44	10.80	11.21	0.79
YBOX1_MOUSE	0.164124	16.68	15.95	16.70	15.46	16.04	16.29	0.78
NACA_MOUSE	0.164437	14.53	14.47	14.50	14.01	13.06	14.55	0.78
NACAM_MOUSE	0.164437	14.53	14.47	14.50	14.01	13.06	14.55	0.78
CNPY2_MOUSE	0.17626	12.46	12.99	10.04	13.61	13.63	12.29	0.75
RS25_MOUSE	0.183649	14.17	15.71	15.46	15.74	16.79	15.35	0.74
ERD21_MOUSE	0.186208	13.46	13.59	12.78	14.44	13.81	13.28	0.73
PSMD7_MOUSE	0.187718	12.96	13.17	10.64	11.64	9.38	11.52	0.73
1433T_MOUSE	0.192931	14.35	17.16	17.25	17.07	17.93	17.52	0.71
AIMP2_MOUSE	0.19423	10.62	11.43	10.63	10.92	9.42	10.34	0.71
1433F_MOUSE	0.201946	10.88	16.87	16.97	16.94	17.85	17.47	0.69
SCOT1_MOUSE	0.206602	13.33	15.39	15.53	13.71	12.49	14.59	0.68
1433B_MOUSE	0.2371	15.42	17.34	17.39	17.11	17.88	17.52	0.63
NPHP3_MOUSE	0.243422	15.80	15.77	14.74	13.76	15.10	15.37	0.61
RL30_MOUSE	0.247419	17.16	15.05	17.18	15.81	15.95	15.10	0.61
ELOV5_MOUSE	0.258405	13.97	13.78	13.99	13.88	13.83	12.76	0.59
NPC2_MOUSE	0.259318	11.08	11.64	12.19	11.76	10.87	10.79	0.59
DNPEP_MOUSE	0.264156	13.89	11.40	13.97	13.04	9.50	12.33	0.58
ARSA_MOUSE	0.264454	13.47	10.82	11.47	10.86	11.57	10.60	0.58
TPM3_MOUSE	0.266344	17.81	17.98	17.64	18.06	18.04	17.89	0.57
RS5_MOUSE	0.266611	17.31	17.41	17.23	17.32	17.65	17.53	0.57
RS13_MOUSE	0.285418	13.47	14.36	13.52	12.41	13.83	13.49	0.54
PUR9_MOUSE	0.285772	10.00	8.60	10.64	9.62	8.78	8.79	0.54
PTK6_MOUSE	0.28915	0.00	0.00	0.00	0.00	0.00	9.70	0.54
K2C79_MOUSE	0.289352	18.86	19.12	18.84	18.83	19.52	19.25	0.54
K2C7_MOUSE	0.292854	15.60	15.21	15.45	15.42	15.55	16.08	0.53
GFAP_MOUSE	0.292854	15.60	15.21	15.45	15.42	15.55	16.08	0.53
KRT85_MOUSE	0.292854	15.60	15.21	15.45	15.42	15.55	16.08	0.53
VATE1_MOUSE	0.298284	12.38	12.47	12.01	12.23	12.30	10.48	0.53
UNC79_MOUSE	0.301243	9.70	11.27	0.00	10.56	10.17	10.54	0.52
TPM1_MOUSE	0.310726	17.63	17.77	17.32	17.80	17.70	17.76	0.51
ROAA_MOUSE	0.312399	17.53	17.26	17.46	17.13	17.28	17.35	0.51
TCPQ_MOUSE	0.328145	14.84	13.00	14.61	12.67	13.87	13.94	0.48
IPO4_MOUSE	0.330746	10.94	12.57	12.78	11.74	11.10	11.72	0.48
RB11A_MOUSE	0.332879	12.52	12.83	10.92	11.77	11.88	10.66	0.48
FUS_MOUSE	0.334276	14.99	14.25	14.38	14.36	14.39	14.12	0.48
RL9_MOUSE	0.335137	11.52	14.10	14.36	11.45	12.84	12.90	0.47
PSME2_MOUSE	0.345432	10.59	12.14	12.92	12.12	12.94	12.54	0.46
RL22_MOUSE	0.356258	14.62	15.45	14.44	14.52	14.64	14.47	0.45
H2AX_MOUSE	0.363435	19.34	19.36	18.97	19.20	19.02	18.95	0.44
PGK1_MOUSE	0.365098	17.57	17.47	17.76	17.39	17.38	17.60	0.44
ACTB_MOUSE	0.372193	16.23	17.58	17.39	16.60	17.10	16.23	0.43
RS15_MOUSE	0.373532	8.56	11.70	9.45	11.67	9.21	12.03	0.43
H2A2B_MOUSE	0.375984	19.31	19.32	18.93	19.14	18.99	18.94	0.42
TCTP_MOUSE	0.389556	15.70	16.11	16.01	16.15	15.85	16.41	0.41
K2C73_MOUSE	0.394735	17.48	17.28	17.24	17.18	16.86	17.43	0.40
1433G_MOUSE	0.397852	16.37	17.70	17.82	17.36	18.05	17.71	0.40
NDKB_MOUSE	0.400574	15.12	15.57	15.59	15.81	15.35	15.69	0.40
RLA1_MOUSE	0.409863	18.51	18.47	18.84	18.63	18.76	18.85	0.39
ABCE1_MOUSE	0.411465	14.21	12.17	14.39	13.79	11.70	13.11	0.39
PUR4_MOUSE	0.415174	13.27	13.77	12.35	13.18	13.17	11.32	0.38
H2AJ_MOUSE	0.419493	19.30	19.32	18.91	19.17	18.99	18.92	0.38
H2A1K_MOUSE	0.419493	19.30	19.32	18.91	19.17	18.99	18.92	0.38
H2A1H_MOUSE	0.419493	19.30	19.32	18.91	19.17	18.99	18.92	0.38
H2A1F_MOUSE	0.419493	19.30	19.32	18.91	19.17	18.99	18.92	0.38
H2A3_MOUSE	0.419493	19.30	19.32	18.91	19.17	18.99	18.92	0.38
H2A1P_MOUSE	0.419493	19.30	19.32	18.91	19.17	18.99	18.92	0.38
H2A1O_MOUSE	0.419493	19.30	19.32	18.91	19.17	18.99	18.92	0.38
H2A1N_MOUSE	0.419493	19.30	19.32	18.91	19.17	18.99	18.92	0.38
H2A1I_MOUSE	0.419493	19.30	19.32	18.91	19.17	18.99	18.92	0.38
H2A1G_MOUSE	0.419493	19.30	19.32	18.91	19.17	18.99	18.92	0.38
H2A1E_MOUSE	0.419493	19.30	19.32	18.91	19.17	18.99	18.92	0.38
H2A1D_MOUSE	0.419493	19.30	19.32	18.91	19.17	18.99	18.92	0.38
H2A1C_MOUSE	0.419493	19.30	19.32	18.91	19.17	18.99	18.92	0.38
H2A1B_MOUSE	0.419493	19.30	19.32	18.91	19.17	18.99	18.92	0.38
H2A2A_MOUSE	0.436243	19.27	19.28	18.86	19.11	18.96	18.91	0.36
H2A2C_MOUSE	0.436243	19.27	19.28	18.86	19.11	18.96	18.91	0.36
RS17_MOUSE	0.439512	13.88	10.54	13.59	13.79	12.66	14.01	0.36
LYAG_MOUSE	0.450325	12.54	9.97	11.66	10.70	10.94	10.93	0.35
THOC4_MOUSE	0.462349	14.38	14.69	14.65	14.27	14.65	14.39	0.34
ALRF2_MOUSE	0.462349	14.38	14.69	14.65	14.27	14.65	14.39	0.34
NDUA4_MOUSE	0.471139	13.69	12.04	13.71	12.39	12.33	13.40	0.33
AT5F1_MOUSE	0.482549	16.29	12.89	14.77	12.77	14.33	14.64	0.32
RM12_MOUSE	0.506745	13.70	13.65	12.79	13.52	13.33	12.46	0.30
RCN2_MOUSE	0.518016	14.14	13.87	13.66	13.93	13.85	14.32	0.29
RS10_MOUSE	0.52846	11.58	14.25	14.08	12.52	13.43	12.35	0.28
RL31_MOUSE	0.540627	16.99	16.90	15.24	15.88	16.37	15.86	0.27
RS18_MOUSE	0.556426	12.92	12.59	12.58	12.37	12.42	12.91	0.25
TCEA1_MOUSE	0.565193	12.59	14.22	13.07	13.90	10.80	13.47	0.25
TMED9_MOUSE	0.58254	12.07	12.94	9.11	13.17	11.80	11.11	0.23
GSTP2_MOUSE	0.589048	16.49	14.57	15.66	15.38	15.37	15.13	0.23
GSTP1_MOUSE	0.589048	16.49	14.57	15.66	15.38	15.37	15.13	0.23
SKP1_MOUSE	0.610602	12.96	15.48	15.74	15.01	15.06	15.35	0.21
QCR2_MOUSE	0.618647	10.32	9.89	11.16	11.05	9.97	9.53	0.21
AQP1_MOUSE	0.642568	8.72	9.75	11.31	9.64	11.31	9.98	0.19
MTPN_MOUSE	0.674227	13.79	14.86	14.62	14.51	14.60	13.64	0.17
ARL1_MOUSE	0.677382	14.41	13.58	14.00	14.95	14.22	13.41	0.17
SCRB2_MOUSE	0.678152	12.27	11.87	11.01	11.23	12.12	11.24	0.17
K2C1B_MOUSE	0.689647	18.32	18.49	18.36	18.23	18.37	18.80	0.16
PTMA_MOUSE	0.696432	21.09	21.06	21.09	21.06	21.37	20.99	0.16
1433E_MOUSE	0.697313	18.88	19.57	19.80	19.17	19.76	18.90	0.16
TPM4_MOUSE	0.7118	19.25	19.19	18.96	18.93	19.47	19.23	0.15
S10AA_MOUSE	0.715815	12.43	14.11	14.26	13.96	13.71	13.74	0.15
MARCS_MOUSE	0.726697	14.39	14.24	13.95	14.54	14.64	13.72	0.14
CAPR1_MOUSE	0.731991	12.49	13.16	13.04	12.61	12.96	13.42	0.14
SET_MOUSE	0.745103	17.88	18.09	18.00	17.96	18.02	17.86	0.13
PLP2_MOUSE	0.748147	11.74	13.38	12.77	13.13	12.22	12.02	0.13
ATPD_MOUSE	0.758636	11.61	12.28	12.48	11.64	13.01	11.20	0.12
PUSL1_MOUSE	0.767776	10.10	12.69	12.69	9.81	11.75	12.92	0.11
ACTY_MOUSE	0.782008	11.23	13.96	14.26	13.44	13.43	13.30	0.11
ACTZ_MOUSE	0.782008	11.23	13.96	14.26	13.44	13.43	13.30	0.11
DBF4A_MOUSE	0.788214	12.57	0.00	0.00	0.00	0.00	17.81	0.10
GPM6A_MOUSE	0.809664	18.65	18.79	18.22	19.09	18.01	18.80	0.09
PSME1_MOUSE	0.842895	12.30	13.00	13.15	12.88	13.04	12.70	0.07
COX41_MOUSE	0.930115	11.07	14.37	14.74	12.28	13.97	13.62	0.03
PP14B_MOUSE	0.932589	12.48	12.62	12.40	12.41	12.79	12.25	0.03
CO6A1_MOUSE	0.945765	9.84	11.39	10.75	12.41	9.66	9.73	0.02
CO1A2_MOUSE	0.94961	11.48	14.35	13.89	12.85	13.72	13.31	0.02
HARS1_MOUSE	0.960346	9.84	10.24	11.74	11.12	10.46	10.32	0.02
CALR_MOUSE	0.967791	16.10	16.32	16.31	16.07	15.89	16.80	0.01
BAP31_MOUSE	0.973337	10.98	13.81	13.19	12.35	13.30	12.25	0.01
EI3JB_MOUSE	0.97778	12.28	12.65	11.10	10.89	12.88	12.31	0.01
EI3JA_MOUSE	0.97778	12.28	12.65	11.10	10.89	12.88	12.31	0.01
MIF_MOUSE	0.98048	18.20	18.05	18.36	17.61	19.16	17.87	0.01
