# Supplementary material for: Study of the Lipolysis Effect of Nanoliposome-Encapsulated Ganoderma lucidum Protein Hydrolysates on Adipocyte Cells Using Proteomics Approach
Source: Foods. 2021 Sep 12;10(9):2157. doi: 10.3390/foods10092157 (PMC8468392; doi:10.3390/foods10092157)
Supplement: Supplementary file 1 [file foods-10-02157-s001.zip › Supplemetary figures.pdf]

Supplementary Figures

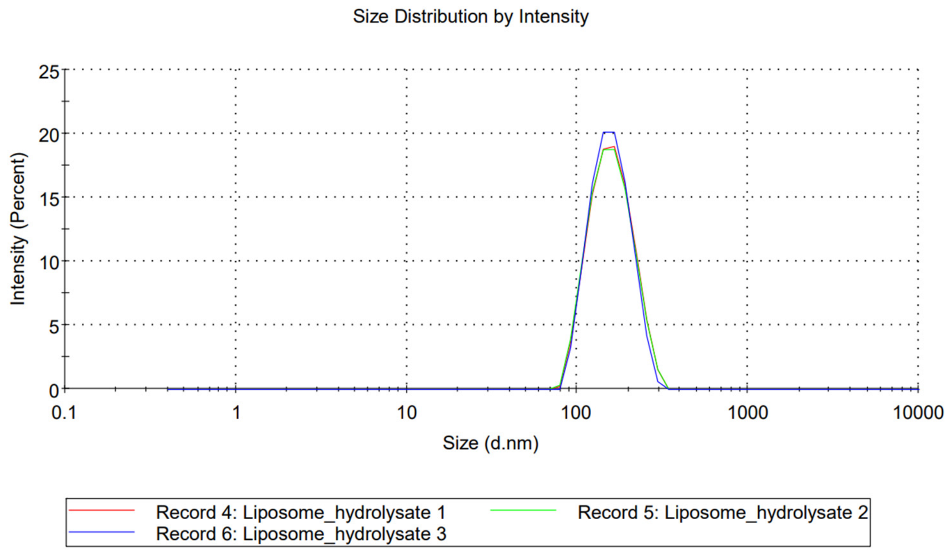

Figure S1: Dynamic light scattering (DLS) analysis showed size distribution of loaded liposome particle at 25 °C in the PBS solution (n=3).

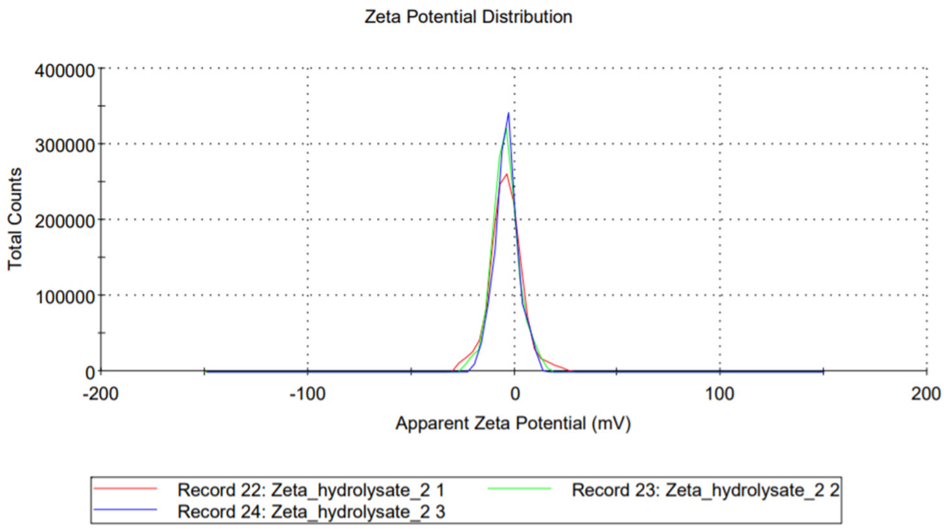

Figure S2: Dynamic light scattering (DLS) analysis showed zeta-potential distribution of loaded liposome particle at 25 °C in the PBS solution (n=3).

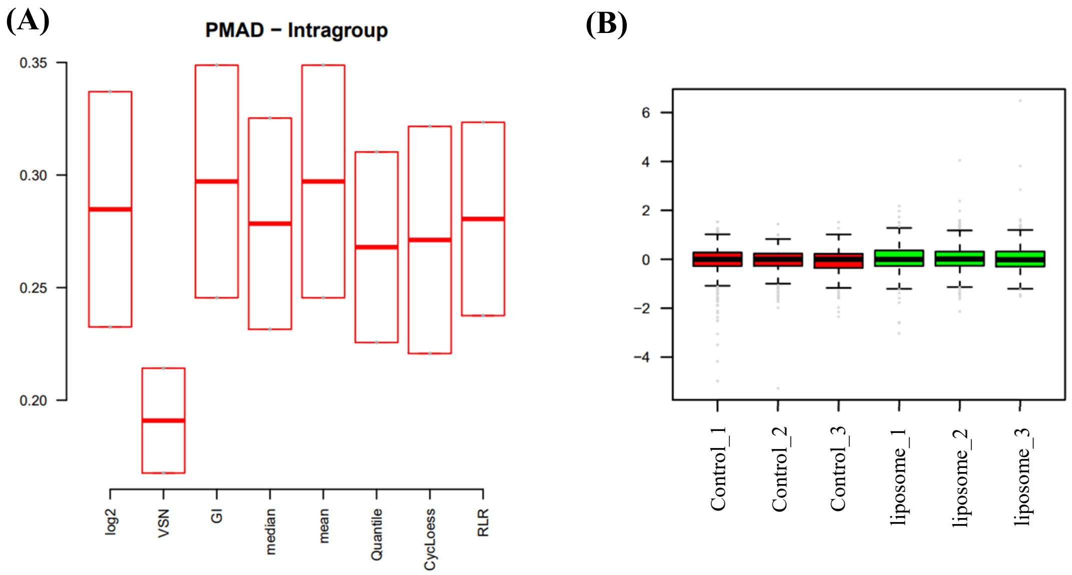

Supplementary Figure S3: Intragroup variation was measured with pooled intragroup median absolute deviation (PMAD) of the identified proteins (A) and Relative Log Expression (RLE) plots (B).
